# Risk of venous thromboembolism after total hip and knee replacement in older adults with comorbidity and co-occurring comorbidities in the Nationwide Inpatient Sample (2003-2006)

**DOI:** 10.1186/1471-2318-10-63

**Published:** 2010-09-17

**Authors:** Alok Kapoor, Alan J Labonte, Michael R Winter, Jodi B Segal, Rebecca A Silliman, Jeffrey N Katz, Elena Losina, Dan Berlowitz

**Affiliations:** 1 Hospital Medicine Unit, Boston University School of Medicine, 715 Albany Street, Boston, 02118, MA, USA; 2 Data Coordinating Center, Boston University School of Public Health, 715 Albany Street, Talbot Building, Boston, 02118, MA, USA; 3 Department of Medicine, Johns Hopkins University School of Medicine, 733 N. Broadway, Baltimore, 21205, MD, USA; 4 Section of Geriatrics, Boston University School of Medicine, 715 Albany Street, Boston, 02118 MA, USA; 5 Section of Epidemiology, Boston University School of Public Health, 715 Albany Street, Talbot Building, Boston, 02118, MA, USA; 6 Department of Orthopaedic Surgery and Division of Rheumatology, Immunology, and Allergy, Orthopaedic and Arthritis Center for Outcomes Research, Brigham and Women's Hospital, 75 Francis Street, Boston, 02115, MA, USA; 7 Harvard Medical School, 25 Shattuck Street, Boston, 02115, MA, USA; 8 Department of Orthopaedic Surgery, Brigham and Women's Hospital, 75 Francis Street, Boston, 02115, MA, USA; 9 Department of Biostatistics, Boston University School of Public Health, 715 Albany Street, Talbot Building, Boston, 02118, MA, USA; 10 Center for Health Quality, Outcomes, and Economic Research, Edith Nourse Rogers Memorial VA Hospital, 200 Springs Road, Bedford, 01730, MA, USA; 11 Boston University School of Public Health 715 Albany Street, Talbot Building, Boston, 02118, MA, USA

## Abstract

**Background:**

Venous thromboembolism is a common, fatal, and costly injury which complicates major surgery in older adults. The American College of Chest Physicians recommends high potency prophylaxis regimens for individuals undergoing total hip or knee replacement (THR or TKR), but surgeons are reluctant to prescribe them due to fear of excess bleeding. Identifying a high risk cohort such as older adults with comorbidities and co-occurring comorbidities who might benefit most from high potency prophylaxis would improve how we currently perform preoperative assessment.

**Methods:**

Using the Nationwide Inpatient Sample, we identified older adults who underwent THR or TKR in the U.S. between 2003 and 2006. Our outcome was VTE, including any pulmonary embolus or deep venous thrombosis. We performed multivariate logistic regression analyses to assess the effects of comorbidities on VTE occurrence. Comorbidities under consideration included coronary artery disease, congestive heart failure (CHF), chronic obstructive pulmonary disease (COPD), diabetes, and cerebrovascular disease. We also examined the impact of co-occurring comorbidities on VTE rates.

**Results:**

CHF increased odds of VTE in both the THR cohort (OR = 3.08 95% CI 2.05-4.65) and TKR cohort (OR = 2.47 95% CI 1.95-3.14). COPD led to a 50% increase in odds in the TKR cohort (OR = 1.49 95% CI 1.31-1.70). The data did not support synergistic effect of co-occurring comorbidities with respect to VTE occurrence.

**Conclusions:**

Older adults with CHF undergoing THR or TKR and with COPD undergoing TKR are at increased risk of VTE. If confirmed in other datasets, these older adults may benefit from higher potency prophylaxis.

## Background

Medical injury, or harm associated with a therapeutic or diagnostic intervention [1], commonly complicates major surgery [2]. Age alone confers a small effect on postoperative injury but medical comorbidities and co-occurring comorbidities contribute substantially. Co-occurring comorbidities, that is, 2 comorbidities which frequently occur together, are part of a larger phenomenon of multiple morbidity [3].

Venous thromboembolism (VTE) is a common, often fatal, and costly injury which complicates major surgery in older adults. VTE is particularly common following total hip and knee replacement (THR and TKR), with venographic rates of up to 60% without prophylaxis [4]. Newer, more potent prophylaxis regimens including the synthetic pentasacchride fondaparinux and twice daily dosing of enoxaparin, a low molecular weight heparin, offer the ability to significantly reduce the risk of VTE. Their wide spread adoption has been slow, however, given the increased risk of bleeding [5]. The ability to identify a high risk cohort among older adults undergoing THR and TKR, who would potentially benefit from high potency prophylaxis, would be an improvement in the way clinicians currently conduct preoperative assessments.

Comorbidities such as congestive heart failure (CHF) and chronic obstructive pulmonary disease (COPD) have been associated with increased VTE risk in some studies [6-8] but not in others [9,10]. The association with CHF may relate to blood flow stasis which is part of Virchow's triad [11] of alteration in blood flow (stasis), endothelial injury, and alterations in blood constituents (hypercoagulable factors). Similarly the association of COPD may be explained through an immobility or stasis mechanism. Newer evidence, in both surgical [6,7,9,10] and non-surgical settings [12], suggests that atherosclerotic conditions such as coronary artery disease (CAD) and cerebrovascular disease (CVD) are also associated with an increased risk of VTE. In general, these studies had a small number of observations and/or did not focus on discrete surgical procedures, making it difficult to draw conclusions about modest but clinically important effects on VTE of comorbidities for individual surgeries. The combined effect of comorbidities, i.e., co-occurring comorbidities, has been incompletely evaluated.

In this investigation, we assessed the Nationwide Inpatient Sample to determine the risk of VTE in older adults with prevalent comorbidities and co-occurring comorbidities undergoing THR or TKR. We hypothesized that CHF, CAD, CVD, and COPD would predict increased risk of VTE. We also proposed that the joint effect of co-occurring comorbidities -- composed of comorbidities with distinct pathogenesis, such as CAD and COPD -- would equal the sum of the component effects or possibly exceed their sum, indicating a positive interaction.

## Methods

### Data Sources and Study Sample

We used the Nationwide Inpatient Sample (NIS) from 2003-2006 for our analysis [13]. This work involves the use of publicly available, archival information abstracted from patient medical records and was approved by the Institutional Review Board at the Boston University Medical Center in Boston, Massachusetts. The NIS contains information from hospital inpatient stays collected at the state-level and then assembled by the Agency for Healthcare Research and Quality (AHRQ) as part of the Healthcare Utilization Project (HCUP). NIS includes primary and secondary diagnoses, procedures, admission and discharge status, patient demographics (gender, age, race, median income, and residence zip code), expected payment source, total charges, length of stay, and hospital characteristics (ownership, size, and teaching status). We identified a cohort of patients (age 65 or older) who underwent primary THR or TKR identified through ICD-9-CM procedure codes 81.51 and 81.54. We restricted our analysis to elective, primary THR and TKR because we felt that non-elective surgery and revision joint surgery represented a distinct population with different risks for VTE. We therefore excluded surgical cases conducted in the setting of fracture, pelvic or thigh infection, or removal of prior prosthesis, internal fixation device, implant or graft consistent with earlier work [14,15] conducted by members of our team.

### Outcomes and Exposures

We determined VTE to have occurred if any one of eight ICD-9-CM DVT codes, three PE codes, and two non-specific VTE codes were present at discharge (Additional file 1).

We included specific comorbidities based on evidence of associations with VTE documented in the literature or a known biological link to the outcomes These included CAD, CHF, COPD, CVD, and diabetes. Comorbidities and presence of obesity were coded as by Elixhauser et al.[16] (Additional file 1). Multiple publications [17-19] have supported the use of the Elixhauser comorbidity coding algorithms rather than the earlier Charlson comorbidity index, including those looking at discrete surgical procedures [20,21]. The accuracy of coding of the comorbidities has been validated in multiple publications [22,23]. We also examined co-occurring comorbidities from the above mentioned comorbidities if they had a prevalence of greater than 2% (given that exposures occurring less frequently than 2% of the time would be infeasible to study because of sample size constraints).

Consistent with previous work [14,15,24], we evaluated the effects of potentially confounding factors, i.e., race, insurance status, hospital surgical volume, obesity, bi-laterality (two primary procedures during the same surgery), chronic kidney disease, and hypercoagulable state (including cancer and genetic predisposition), by adding them, one at a time, to the crude model (with comorbidity exposure, age group, and sex). If the addition of a given factor did not change the odds ratios for the comorbidity exposure by more than 10%, it was deleted from the final model.

### Analysis

We calculated descriptive statistics and cross-tabular frequencies for each outcome and comorbidity to determine the shape of distributions, the extent of missing data, and the presence of small frequencies of the discrete comorbidity levels. We examined comorbidities for statistical interactions with each other. Specifically, we measured whether prevalent combinations of comorbidities (as defined above) produced statistically significant associations with VTE in multivariate models. Because we found these to be present in the TKR cohort, for the ease of interpretability, we report the associations between co-occurring comorbidities and outcomes using a categorical comorbidity variable for both the THR and TKR cohorts. We created a ten level categorical variable with a separate, mutually exclusive, level for each of nine comorbidities or co-occurring comorbidities (with 1 additional for all other combinations of two or more comorbidities). While this limited the population size for each comorbidity group, it allowed us to compare the risk of VTE for groups of older adults with single or co-occurring comorbidities against a common reference group of older adults without any of the nine comorbidities.

Finally, we built multivariate logistic regression models to assess the independent effects of comorbidities. In our analysis we retained age and sex in all models. For remaining potential confounders, we retained any variable that changed the effect estimate for any comorbidity by more than 10%.

## Results

In NIS 2003-2006, we identified 93,071 primary THR and 223,600 primary TKR surgeries. Overall, the age of older adults was evenly distributed into quartiles 65-69, 70-74, 75-79, and 80 or older. Sixty-three percent of subjects were female. (Table 1) CAD, COPD, and diabetes alone occurred most frequently (9.8%, 8.6%, and 8.4%, respectively for the THR cohort). CVD and CHF occurred much less frequently (0.6% and 1.2%, respectively for the THR cohort). (Table 1) CAD with diabetes was present in 2.5%, and CAD with COPD was present in 1.9% of the THR cohort. (Table 1) Other combinations occurred approximately 1% of the time. Similar trends occurred in the TKR cohort.

**Table 1:** Frequency counts (and row percentages) for selected variables and stratified by surgical procedure.

	Total Hip Replacement	Total Knee Replacement
**Age**		
65-69 yr	23405 (25.2)	63363 (28.3)
70-74 yr	24888 (26.7)	63608 (28.5)
75-79 yr	23316 (25.0)	54578 (24.4)
≥80 yr	21462 (23.1)	42051 (18.8)
		
**Gender**		
Male	34578 (37.2)	78539 (35.1)
Female	58492 (62.8)	145061 (64.9)
		
**Comorbid Diseases**		
Cerebrovascular Disease (CVD) alone	570 (0.6)	1356 (0.6)
Coronary Artery Disease (CAD) alone	9132 (9.8)	20017 (9.0)
Congestive Heart Failure (CHF) alone	1097 (1.2)	2746 (1.2)
Diabetes alone	7782 (8.4)	26740 (12.0)
Chronic Obstructive Pulmonary Disease (COPD) alone	7989 (8.6)	16836 (7.5)
CAD and CHF alone	731 (0.8)	1678 (0.8)
CAD and COPD alone	1731 (1.9)	3366 (1.5)
CAD and Diabetes alone	2320 (2.5)	7155 (3.2)
COPD and Diabetes alone	1181 (1.3)	3688 (1.7)
All other 2+ combinations	2725 (2.9)	6878 (3.1)
None of the above comorbidities	57813 (62.0)	133140 (59.4)
		
**Median Length of Stay **(days)	4	3

VTE during the index hospitalization occurred 0.8% of the time after THR and 1.2% of the time after TKR. In the THR cohort, the rate of VTE ranged from 0.6% in older adults with diabetes alone to 2.3% in older adults with CHF alone; the rate of VTE in older adults without any of the identified comorbidities was 0.7%. (Table 2) Similarly, in the TKR cohort, this range was 1.1% to 2.6%; the rate of VTE in older adults without any of the identified comorbidities was 1.1%. In multivariate analysis CHF predicted a threefold increase in the odds of VTE in the THR cohort (OR = 3.08 95% CI 2.05-4.65) and a similar increase in the TKR cohort (OR = 2.47 95% CI 1.95-3.14). (Table 3) COPD predicted a 50% increase in odds in the TKR cohort (OR = 1.49 95% CI 1.31-1.70). (Table 3)

**Table 2:** Frequency counts for selected variables stratified by age and surgical procedure.

	Age Group									
	Hip Procedures					Knee Procedures				
Exposure	65-69 years of age	70-74 years of age	75-79 years of age	≥80 years of age	total	65-69 years of age	70-74 years of age	75-79 years of age	≥80 years of age	total
CVD alone	87	129	160	194	570	241	342	382	391	1356
CAD alone	1677	2269	2503	2683	9132	4065	5413	5608	4931	20017
CHF alone	143	209	277	468	1097	441	574	702	1029	2746
Diabetes alone	2159	2229	1935	1459	7782	8975	8197	6116	3452	26740
COPD alone	2110	2220	1949	1710	7989	4978	4873	4065	2920	16836
CAD and CHF alone	87	124	190	330	731	249	335	449	645	1678
CAD and COPD alone	359	443	485	444	1731	726	937	978	725	3366
CAD and Diabetes alone	512	673	632	503	2320	1909	2216	1906	1124	7155
COPD and Diabetes alone	341	356	298	186	1181	1359	1148	772	409	3688
All other 2+ combinations	482	673	763	807	2725	1593	1904	1896	1485	6878
None of the above comorbidities	15448	15563	14124	12678	57813	38827	37669	31704	24940	133140

**Abbreviations**: CAD = coronary artery disease, CHF = congestive heart failure, COPD = chronic obstructive pulmonary disease, CVD = cerebrovascular disease.

**Table 3:** Odds ratios (and 95% CIs) for exposures, adjusted^† ^for covariates and stratified by surgical procedure.

	HIP PROCEDURES				KNEE PROCEDURES			
Exposure	VTE yes	VTE no	**OR**^†^	CIs	VTE yes	VTE no	OR	CIs
No Comorbidity	410	57403	**1.00**	**(ref)**	1458	131682	**1.00**	**(ref)**
CVD vs.	3	567	**0.71**	(0.23-2.23)	21	1335	**1.42**	(0.92-2.20)
CAD vs.	68	9064	**0.98**	(0.76-1.28)	257	19760	**1.19**	(1.04-1.37)
CHF vs.	25	1072	**3.08**	(2.05-4.65)	73	2673	**2.47**	(1.95-3.14)
Diabetes vs.	47	7735	**0.85**	(0.63-1.15)	282	26458	**0.96**	(0.85-1.10)
COPD vs.	60	7929	**1.06**	(0.81-1.39)	273	16563	**1.49**	(1.31-1.70)
CAD and CHF vs.	12	719	**2.11**	(1.18-3.78)	33	1645	**1.86**	(1.30-2.60)
CAD & COPD vs.	16	1715	**1.25**	(0.75-2.06)	52	3314	**1.44**	(1.09-1.90)
Diabetes & CAD vs.	18	2302	**1.05**	(0.65-1.69)	47	7108	**0.61**	(0.45-0.81)
Diabetes & COPD vs.	9	1172	**1.09**	(0.56-2.11)	36	3652	**0.89**	(0.64-1.24)
All other combinations	34	2691	**1.69**	(1.19-2.40)	111	6767	**1.49**	(1.23-181)
Age 65-69	163	23242	**1.00**	**(ref)**	734	62629	**1.00**	**(ref)**
Age 70-74 vs. Age 65-69	167	24721	**0.96**	(0.77-1.19)	751	62857	**1.01**	(0.91-1.12)
Age 75-79 vs. Age 65-69	172	23144	**1.05**	(0.84-1.30)	649	53929	**1.00**	(0.90-1.12)
Age ≥80 vs. Age 65-69	200	21262	**1.30**	(1.05-1.60)	509	41542	**0.99**	(0-89-1.11)
Females	411	58081	**1.00**	**(ref)**	1746	143315	**1.00**	**(ref)**
Males	291	34287	**1.21**	(1.04-1.41)	897	77642	**0.94**	(0.87-1.02)
Total observations	702	92369			2643	220957		

^†^Associations adjusted in multivariate logistic regression for each comorbidity exposure as compared with the group with none of the listed comorbidities or co-occurring comorbidities. All association were adjusted for age group and sex. We also assessed race, insurance status, hospital surgical volume, obesity, bi-laterality (two primary procedures during the same surgery) and hypercoagulable state but deleted them from the final model for lack of confounding. Abbreviations: CAD = coronary artery disease, CHF = congestive heart failure, COPD = chronic obstructive pulmonary disease, CVD = cerebrovascular disease.

We did not find any positive interactions between comorbidities in our analysis. The combination of CAD and CHF alone was associated with a twofold increase in odds in both the THR and TKR cohort, an effect which was smaller than for CHF alone. The combination of diabetes and CAD was associated with a 39% decrease in the odds of VTE only in the TKR cohort, an effect which was smaller, i.e., more protective, than either comorbidity alone. (Table 3) The six potential confounding factors that we tested were deleted from the final model for lack of confounding.

## Discussion

We comprehensively examined the association of comorbidities and co-occurring comorbidities and VTE in a unique population of older adults undergoing primary total hip and knee replacement, high risk surgeries for VTE. We found that the rates of VTE captured in administrative data of older adults for the period immediately following THR and TKR to be low at 0.8-1.2%. Having CHF substantially increased the odds of VTE after THR or TKR and having COPD somewhat increased the odds of VTE after TKR. Co-occurring comorbidities did not increase the risk of VTE beyond their individual effects.

Comparison of our results with previous studies is limited by differences in outcomes measured and populations studied. Gangireddy et al. [9] conducted one of the largest studies to date using data from the Veterans Affairs National Surgical Quality Improvement Program (NSQIP), which included veterans undergoing nine different surgeries, including THR, between 1996 and 2001. After controlling for multiple preoperative and postoperative clinical variables, a multivariate analysis with 76,771 individuals showed that CHF and COPD were not associated with increased rates of VTE. This study supported our findings of the association between diabetes and a slightly lower rate of VTE (OR = 0.75).

We also detected a 29% reduction in the risk of VTE in patients undergoing THR with CVD. This result was not statistically significant nor did we detect an association in the knee population. There has been little evidence, however, regarding the relationship between CVD and postoperative VTE. Prior work [12] has suggested a common inflammatory pathway but this has not been evaluated extensively in the postoperative setting. In our study the association was not present for both knee and hip cohorts and was not statistically significant. We plan to re-assess this relationship in our future work.

Kikura et al. [6] examined 21,903 Japanese patients of multiple ages and multiple surgery types and found that history of acute myocardial infarction (AMI) was significantly related (OR = 7.7 95% CI 1.7-34.7) to the development of postoperative thrombotic events (including repeat AMI). Although we did collect information about the history of AMI in particular, we did not find an association with CAD in general in our analysis. In a cohort of 269 post-menopausal women undergoing THR and TKR, Jaffer et al. [7] found a trend towards CHF predicting more postoperative VTE events (OR = 5.50 95% CI 0.94-43.3) but a trend towards COPD predicting fewer VTE events (OR = 0.42 95% CI 0.07-1.98).

The association of CHF and VTE may relate to blood flow stasis as discussed earlier. Alternatively, CHF may indicate a degree of immobility that was not measured in the data we analyzed. Other comorbidities may also contribute to the development of postoperative VTE but their effects may have been attenuated by a selection bias. Surgeons may select only the healthiest subset of older adults with comorbidities for surgery. The absence of positive interactions between frequently co-occurring comorbidities (especially CAD and CHF) also suggests a potential source of a selection bias. Older adults with co-occurring comorbidities deemed to be suitable surgical candidates are presumably healthier in other ways than other older adults with the same comorbidities.

In the case of COPD, we only detected an increase in risk for older adults undergoing knee surgery. This could be explained by the generally weak predictor effect of COPD on VTE or it could be related to the inherent differences between hip and knee surgery. Postoperative mobility may be significantly less for hip surgery and the effect of immobility in this group may dwarf other predictors such as COPD. Future work should examine the interaction between mobility and surgery type in data where this information is available.

The association of CHF and COPD with postoperative VTE has important implications. Although the American College of Chest Physicians currently recommends high potency prophylaxis such as fondaparinux or LMWH for all individuals undergoing THR and TKR [4], surgeons are reluctant to prescribe these regimens, fearing bleeding complications. Identification of a high risk subset among a group of older adults already at increased risk for VTE based on the surgery planned would be an important improvement in the way we currently perform preoperative assessment. In addition to the use of high potency prophylaxis, surgeons may also use the risk information to incorporate other practices, such as regional anesthesia, mechanical prophylaxis devices, or stockings, aimed at lowering VTE rates.

There are limitations to the work we presented. Due to the nature of the NIS administrative data we have limited ability to capture VTE. A recent study [24], suggests that administrative data capture only 58% of VTE events. There is no evidence, however, to suggest that the events indentified are differentially being diagnosed in individuals with CHF or other comorbidities. In addition, we did not have access to medication information including prophylaxis agent. A recent study by Cohen et al. in 2008 [25] indicated that in the United States, only 48% of medical patients are receiving the recommended ACCP prophylaxis and only 71% of surgical patients are receiving prophylaxis [25]. If comorbidities prompted physicians to prescribe more potent prophylaxis in older adults with CHF or other comorbidities, however, the effects we observed would represent an underestimate of the true effect. A recent survey [26] suggests that orthopedic surgeons vary their prescribing patterns less than 10% of the time when evaluating a patient with cardiopulmonary disease. Future work should examine the relationship between comorbidities and VTE while controlling for prophylaxis agents in data where medication information is available.

We did not have information about events which took place after hospitalization. Given that the median time for development of DVT is 17 days for THR and 7 days for TKR [27] and the median length of stay was 3 or 4 days for each surgery in our analysis, the associations we present may not reflect the experience of older adults who develop injury in the post discharge period. Controlling for length of stay would not disentangle the relation between these comorbidities and VTE and we, therefore, did not control for it in our analysis. Length of stay may very well be a surrogate for immobility and stasis which are on the causal pathway of VTE development. Alternatively, increased length of stay may also be associated with VTE because of added time needed to achieve therapeutic levels of warfarin. Even though NIS data does not allow for measurement of the 30 or 90 day incidence of VTE, we believe that post discharge rates of VTE events will be similarly disparate in individuals with compared to those without comorbidities. In the future we plan to confirm these associations in data where this information is available.

Administrative data are susceptible to upcoding where medical coders assign a diagnosis that may have only been considered but not proven. We did not have information about those VTE events that were present on admission compared to those that occurred during hospitalization. We plan to conduct further validation studies in other databases where pre-existing diagnosis modifiers are available. We cannot firmly establish causality between comorbidities and VTE using the data available to us. Comorbidities may be linked to other processes such as increased operative time or difficulty weaning from a ventilator after surgery. Future research with datasets containing these clinical variables may clarify the exact causal pathway. Lastly, although in our analysis we controlled for the presence of several factors that might increase the risk of VTE, we did not have data on the smoking status of individuals, which might be related to both the exposure and outcome.

## Conclusion

CHF is strongly associated with an increased risk of VTE after THR and TKR; COPD is associated with an increased risk of VTE after TKR. The absence of risk associated with other comorbidities and co-occurring comorbidities may be explained by the selection of healthier older adults for these surgeries or by some of the limiting factors described above. If these findings are confirmed in other datasets, higher risk older adults with comorbidities could potentially benefit from more aggressive preventive interventions including high potency pharmacologic prophylaxis.

## Competing interests

None of the authors have any financial or non-financial competing interests to declare as related to the contents of this manuscript.

## Authors' contributions

AK and AL were responsible for the study design, analysis, interpretation, and manuscript write-up. RAS, JNK, EL, and DB were responsible for the study design, interpretation, and manuscript write-up. MW was responsible for the study analysis and interpretation, and JBS was responsible for the study interpretation and manuscript write-up. All authors read and approved the final manuscript.

## Pre-publication history

The pre-publication history for this paper can be accessed here:

http://www.biomedcentral.com/1471-2318/10/63/prepub

## Supplementary Material

Additional file 1 **Appendix I - ICD-9-CM Procedure and Diagnosis Codes**. total Joint Replacement Procedure Codes and Venous Thromboembolism Diagnosis Codes. Click here for file
