# Supplementary material for: Risk of venous thromboembolism after total hip and knee replacement in older adults with comorbidity and co-occurring comorbidities in the Nationwide Inpatient Sample (2003-2006)
Source: BMC Geriatr. 2010 Sep 17;10:63. doi: 10.1186/1471-2318-10-63 (PMC2949673; doi:10.1186/1471-2318-10-63)
Supplement: Additional file 1 — Appendix I - ICD-9-CM Procedure and Diagnosis Codes. total Joint Replacement Procedure Codes and Venous Thromboembolism Diagnosis Codes. [file 1471-2318-10-63-S1.PDF]

Appendix I – ICD-9-CM Procedure and Diagnosis Codes

Total Joint Replacement Codes

Code	Diagnosis
81.51	Total hip replacement
81.54	Total knee replacement

VTE Codes

ICD-9-CM Pulmonary Embolism Diagnosis Codes

Codes	Diagnosis
4151	Pulmonary Embolism and infarction
41511	Iatrogenic pulmonary embolism and infarction
41519	Iatrogenic pulmonary embolism and infarction

ICD-9-CM Deep Vein Thrombosis Diagnosis codes:

Codes	Diagnosis
45111	Phlebitis and thrombosis of femoral vein (deep) (superficial)
45119	Phlebitis and thrombophlebitis of deep vessel of lower extremities – other
4512	Phlebitis and thrombophlebitis of deep vessel of lower extremities - unspecified
45181	Phlebitis and thrombophlebitis of iliac vein
4519	Phlebitis and thrombophlebitis of other sites – of unspecified sites
45340	DVT-embolism lower ext NOS (OCT 04)
45341	DVT-EMB proximal lower ext (OCT 04)
45342	DVT-EMB distal lower ext (OCT 04)
4538	Other venous embolism and thrombosis of other specified veins
4539	Other venous embolism and thrombosis of unspecified site
